# Optimized Extraction and Characterization of Folates From Date Palm Fruits and Their Tracking During Fruits Wine Fermentation

**DOI:** 10.3389/fnut.2021.699555

**Published:** 2021-09-07

**Authors:** Ziyi Meng, Ling Yi, Qingxin Hu, Zhiyi Lin, Hosahalli S. Ramaswamy, Chao Wang

**Affiliations:** ^1^Department of Food Science and Technology, Jinan University, Guangzhou, China; ^2^Department of Food Science and Agricultural Chemistry, Macdonald Campus of McGill University, Montréal, QC, Canada

**Keywords:** date palm fruits, folate, depectination, wine-making, optimization

## Abstract

Folates belong to the essential B vitamins group and participate in one-carbon metabolism. Date palm fruits (*Phoenix dactilyfera* L. family *Arecaceae*) are consumed by millions of people and are good sources of folates. To date, no detailed study has been carried out on suitable methods for folate extraction from date palm fruits. In the present study, an experimental design using response surface methodology (RSM) was used to maximize the extraction yield of folates from date palm fruits by including enzymatic depectinization. By applying this new strategy and a UHPLC-MS/MS technique for analysis, total folate and different folate vitamers of three cultivars of date palm fruits (Muzafti, Zahdi, and Rubai), brewer's yeast, and fermented date wine were analyzed. The optimized extraction conditions of folates from date palm fruits were found to be a pectinase activity of 47.7 U, an incubation temperature of 40°C, and an incubation time of 38 min, which yielded a total folate content of 191–301 μg/100 g. In brewer's yeast, the extracted total folate content was very high (4,870 μg/100 g), and, in the resulting date wine, it reached a maximum of 700 μg/L on the fifth day. The predominant folate vitamers in date fruit and fruit wine were 5-formyltetrahydrofolate (5-CHO-THF) and 5-methyltetrahydrofolate (5-CH_3_-THF). During date palm fruit fermentation for up to 8 days, the 5-CHO-THF content gradually decreased by 20%, while 5-CH_3_-THF increased linearly from day 1 to day 5 (y = 0.058 x + 0.0284, *R*^2^ = 0.9614). This study shows that date palm fruit and fruit wine are excellent sources of folate, and further study can be focused on different methods to improve folate stability during wine storage.

## Introduction

Folates are water-soluble and essential B group vitamins with important functions in normal growth and development. Folates are involved in the nucleic acid synthesis, repair, and methylation as coenzymes in one carbon transfer metabolic reactions (1). Folate deficiency is still prevalent in South Asia and central and western Africa (2). Folate deficiency can induce several syndromes and diseases such as megaloblastic anemia, neural tube defects, spina bifida, anencephaly, cardiovascular disease, and certain cancers (3).

Folate is essential and the human body is unable to synthesize it, and, therefore, it must be obtained from the dietary sources (4). Typical sources of native folate include leafy green vegetables, legumes, dairy products, liver, citrus fruits, and eggs, with quantities ranging from 50 to 200 μg/100 g (5). The FDA-recommended daily intake is high (400 μg per day), and, hence, identifying some non-traditional food sources rich in natural folate would be useful.

**Figure 1:**
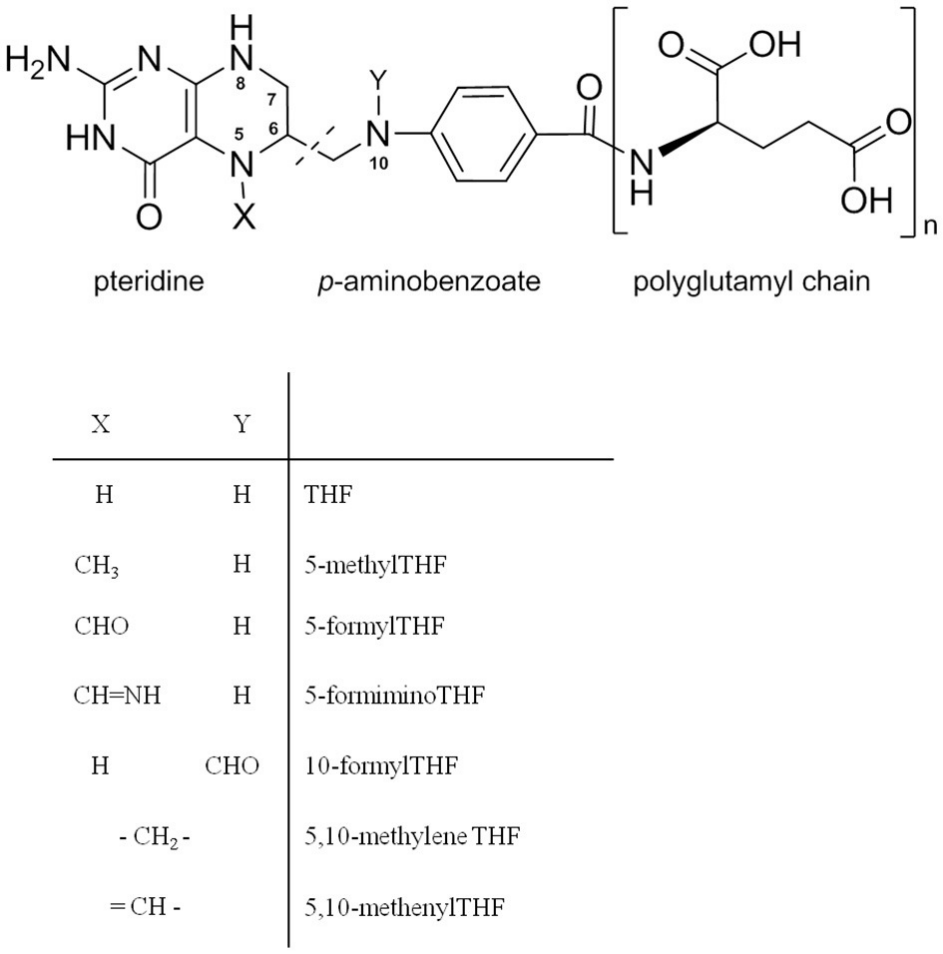
Chemical structures of folate vitamers and polyglutamyl folate.

Chemically, folate is composed of a pteridine ring, *p*-aminobenzoate and is linked to polyglutamyl chains (a variable number of glutamate moieties) (Figure 1) (3). Folate structures differ with the oxidation/reduction state of the pteridine ring, one-carbon substitution at the N5 and N10 positions or numbers of polyglutamyl chain lengths (3). From our previous study, it was found that methyl and formyl substitutions at N5 were the predominant folate forms in common foods which correspond to 5-CH_3_-THF and 5-CHO-THF (6). Folate in food is very susceptible to heat, light, and oxygen (7). Therefore, extraction and determining folate levels in food are very challenging. Several methods have been reported for food folate extraction and determination. For folate extraction, the widely applied method is a trienzyme digestion procedure (α-amylase, protease, and conjugase) (7). The enzymes α-amylase and protease digest the complex carbohydrates and protein structures to release the bound folates. Conjugases further hydrolyze folate polyglutamates to monoglutamates for detection. This method was reported to be particularly effective for cereal-based products; however, this method is not compatible with folate extraction from a high-pectin food matrix. In addition, some previous studies also showed that pectin and oligosaccharides or polysaccharides could entrap and slow the release of folate (8, 9). Therefore, a new strategy needs to be developed to release the entrapped folate in the pectin matrix, which is important for accurate folate identification and quantification. For folate quantification, the microbiological assay is the one most commonly used method. Although this method is adequate for determining total folate content, it cannot distinguish diverse folate vitamers (7). Previously, we developed a UHPLC-MS/MS method for quantification of different folate vitamers in different plants with high sensitivity and accuracy (10).

Date palm (*Phoenix dactilyfera* L. family *Arecaceae*) is an important commercial crop grown in Arab countries and has a delicious sugary taste with a soft chewy texture. Date palm fruits are consumed by millions of people, and, although they are high in calories, they are a good source of several nutrients. The sugar content ranges from 65 to 80% on a dry-weight basis and are mostly present in the inverted form (glucose and fructose). Several studies show that date palm fruits are a pivotal source of dietary fibers, minerals, vitamins, and 23 types of amino acids (some of which are not present in the most popular fruits such as oranges, apples, and bananas) (11–13). They are also rich in antioxidants (14). Date palm fruits are usually consumed as fresh (raw) or milk or in partially dried form as soft fruits, which are more stable. In recent years, date palm fruits have been used for fermentation to make wines, which are also becoming popular (15). Unfortunately, there is a serious dearth of scientific studies on the optimization of folate extraction and quantification of folate vitamers in different varieties of date palm fruits or date palm fruit wines. Since date palm fruits contain a significant content of pectin (0.3%), in our preliminary studies, it was observed that depectination of date palm fruits by pectinase during folate extraction helps to increase the folate recovery yield.

Response surface methodology is an effective experimental, mathematical, and statistical method to optimize an extraction process within a set of ranges for key process variables. Response surface methodology (RSM) can be used to obtain statistical details of the influence of independent variables on the process outcomes and to generate a set of mathematical models that could be used to optimize the process. As compared with orthogonal experimental design, RSM reduces the number of tests but still provides a statistically significant model describing the effect of process variables and their interactions. Such designs have been successfully applied to optimize trienzyme (α-amylase, pronase, and conjugase) extraction of folate from cereals in early reports (16, 17).

Therefore, the objectives of this study were to (a) evaluate and optimize the pectinase-assisted extraction of folates from date palm fruits, using the RSM methodology and the UHPLC-MS/MS method for folate quantification; (b) determine the distribution of folate vitamers and the total folate content in different varieties of dates under optimized conditions; and (c) track changes in folates during the fermentation process for making date palm wine. This is the first detailed study on the optimization of pectinase-assisted extraction of folate from high-sugar date palm fruits and folates in date palm wine.

## Materials and Methods

### Chemicals

All the folate standards employed in this study are shown in Table 1. Liquid chromatography-mass spectrometry (LC-MS) grade water, acetonitrile, and formic acid were obtained from Thermo Fisher Scientific (Fair Lawn, NJ). Ammonium acetate, 2-mercaptoethanol and L-ascorbic acid were purchased from Sigma-Aldrich (Shanghai, China). Klerzyme 150 (pectinase: 3.5 × 10^5^ U/ml, cellulose: 1,875 U/ml) was obtained from DSM (Het Overloon, the Netherlands). Recombinant His-tagged human GGH was obtained from Novus Biologicals (Littleton, CO, USA). All other chemicals were obtained from Schircks Laboratories (Jona, Switzerland), except for 5-CH_3_-[^13^C_5_]Glu·Ca, which was obtained from Merck Eprova AG (Schaffhausen, Switzerland).

**Table 1:** Folate standards used in this study.

	**Standard**	**Abbreviated name**	**Formula**
1	(6R,S)-5-methyl-5,6,7,8-tetrahydrofolic acid, calcium salt	5-CH_3_-Glu.Ca	C_20_H_25_N_7_O_6_.Ca
2	(6S)-5-methyl-5,6,7,8-tetrahydrofolate-[1^3^C_5_]Glu, calcium salt 5MTHF [^13^C_5_] Glu·Ca	5-CH_3_-[^13^C_5_]Glu·Ca	${\text{C}}_{15}^{13}$C_5_H_25_N_7_O_6_·Ca
3	Folic acid	FA	C_19_H_19_N_7_O_6_
4	(6R,S)-5,10-methenyl-5,6,7,8-tetrahydrofolic acid chloride	5,10-CH^+^THF.Cl	C_20_H_22_N_7_O_6_.Cl
5	10-formylfolic acid	10-CHOFA	C_20_H_19_N_7_O_7_
6	(6R,S)-5-formyl-5,6,7,8-tetrahydrofolic acid, calcium salt	5-CHOTHF.Ca	C_20_H_23_N_7_O_7_
7	(6R,S)-5,6,7,8-tetrahydrofolic acid	THF	C_19_H_23_N_7_O_6_

*All were purchased from Schircks Laboratories (Jona, Switzerland) except 5-CH_3_-[^13^C_5_]Glu·Ca, which was from Merck Eprova AG (Schaffhausen, Switzerland)*.

### Date Palm Fruits

**Figure 2:**
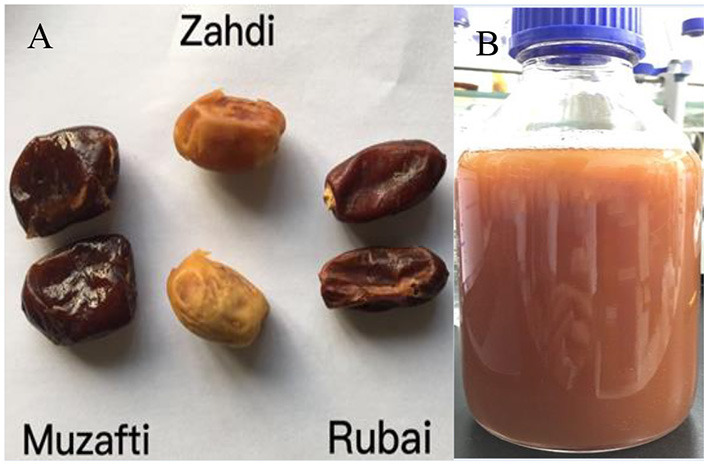
**(A)** Three different cultivars of date palm fruits investigated in this study. **(B)** Date palm fruits wine with cultivar of Rubai.

In this study, three different date palm fruit cultivars were investigated. Pictures of the different date palm fruits are shown in Figure 2A. The cultivars included were Muzafti, Zahdi, and Rubai. All fruits were obtained from Guangdong Wodelong Biotechnology Co., Ltd (Zhuhai, China). The determination of folate vitamers and total folates in date fruits was based on their dry weight without the seeds. Also shown in the Figure 2B, this is the date palm fruit wine with cultivar of Rubai.

### Pectinase Treatment for the Extraction of Folates From Dry Date Palm Fruits

Pectinase treatment was employed as a mean of enhancing the extraction and recovery of folate from date samples since preliminary experiments in our lab demonstrated its advantage. Commercial pectinase (Klerzyme 150, DSM, the Netherland) was used, and, in the first phase, individual factors were evaluated for their influence on folate extraction. The three factors were employed: concentration of pectinase enzyme (10-, 25-, 35-, 50-, and 55-U-per-gram dry fruits), incubation time (0.5, 1, 1.5, 2, and 2.5 h) and incubation temperature (30, 35, 40, 50, and 55°C). For all treatments, the pH of the extract was maintained at 6.

For the extraction process, the dry fruits were gently washed with tap water and surface dried with a paper towel. The seeds were then carefully removed. Samples (5 g) were dispensed into 20 ml of an extraction buffer (100-mM ammonium acetate, 1% ascorbic acid, 0.2% 2-mercaptoethanol, pH7.89) and homogenized for 2 min to a fine puree using a homogenizer (IKA T18 Basic; IKA, Staufen, Germany) at 4°C. The homogenates were immediately boiled for 15 min, cooled on ice to room temperature, brought to 25 ml with distilled water, and then adjusted the pH to 6 for further pectinase treatment. The independent factors of pectinase enzymatic activity (X_1_), incubation temperature (X_2_), and incubation time (X_3_) are listed in Table 2. A stepwise optimization procedure was employed at this stage. All factors were fixed at one of the three levels (−1, 0, 1), with X_1_ (25, 37.5, 50 U/g), X_2_ (30, 35, and 40°C) and X_3_ (0.5, 1, and 1.5 h). To investigate the initial effects of individual factors, one factor was used at different levels, while the others were maintained at specific levels (preselected). For example, for evaluating the temperature effect, the preselected values were 35-U/g enzyme activity and 1-h treatment time. pH was maintained at 6 for all tests. Once the optimum temperature was selected, this value was fixed for testing the next two variables.

**Table 2:** Factors and levels for Box–Behnken design.

**Independent variable**	**Levels**		
	**−1**	**0**	**1**
Pectinase activity (X_1_)	25	37.5	50
Incubation temperature (X_2_)	30	35	40
Incubation time (X_3_)	0.5	1	1.5

Optimization experiments for pectinase release of folate from date palm fruits were then performed. For this, a three-factor-three-level Box–Behnken design (BBD) was employed to obtain the interactions between factors and to define the optimum extraction conditions. The experiment was designed using Minitab 19.0 (State College, Pennsylvania, USA), and the BBD design is shown in Table 3.

**Table 3:** Experimental results for three-level-three-factor Box–Behnken design.

**Run**	**Pectinase activity (X_1_)**	**Incubation temperature (X_2_)**	**Incubation time (X_3_)**	**Total folate (μg/100 g)**		
				**Experimental**	**Predicted**	**Deviation (%)**
1	1	0	1	172.73	172.66	0.04
2	0	0	0	190.83	190.88	0.02
3	0	−1	1	184.16	184.79	0.30
4	−1	−1	0	188.92	188.41	0.27
5	0	0	0	191.15	190.88	0.13
6	0	0	0	190.51	190.88	0.19
7	0	1	−1	187.65	187.14	0.27
8	1	−1	0	183.53	183.09	0.24
9	−1	0	−1	176.86	177.04	0.10
10	−1	1	0	180.67	181.22	0.30
11	−1	0	1	182.57	182.59	0.00
12	1	0	−1	186.70	186.80	0.05
13	0	1	1	174.00	173.63	0.21
14	0	−1	−1	179.40	179.88	0.26
15	1	1	0	185.75	186.37	0.33

Experimental data were fitted for a second-order polynomial model with the following equation:

(1) $$\begin{array}{l} \text{Y}=\;{\text{a}}_{0}+{\text{b}}_{1}{\text{X}}_{1}\;+{\text{b}}_{2}{\text{X}}_{2}+{\text{b}}_{3}{\text{X}}_{3}+{\text{c}}_{12}{\text{X}}_{1}{\text{X}}_{2}+{\text{c}}_{13}{\text{X}}_{1}{\text{X}}_{3}\\ \;+\;{\text{c}}_{23}{\text{X}}_{2}{\text{X}}_{3}+{\text{d}}_{1}{\text{X}}_{1}^{2}+{\text{d}}_{2}{\text{X}}_{2}^{2}+{\text{d}}_{3}{\text{X}}_{3}^{2} \end{array}$$

where Y is the response (total folate content, μg/100 g of the sample), a_0_ represents the interception; b_1_, b_2_, and b_3_ are the linearity; c_12_, c_13_, and c_23_ represent the interaction coefficient; d_1_, d_2_, and d_3_ represent the squared coefficient; and X_1_, X_2_, and X_3_ represent the independent variables.

Analysis of variance was used to determine the individual linear, quadratic, and interaction regression coefficients (β) using Minitab 19.0 (State College, Pennsylvania, USA). The coefficient of determination (*R*^2^) was used to estimate the fitness of the polynomial equation to the response, and the significance of the dependent variables was statistically analyzed by computing the *F-* value at *p* < 0.05.

After the extraction conditions were optimized for the maximum extraction of folate by employing RSM, the responses were experimentally determined under the optimum enzymatic conditions and compared with the predicted values from the RSM model.

### Date Palm Fruit Wine Production

Lab/pilot scale small-volume date palm wine fermentations were carried out in the lab at the Department of Food Science and Technology, Jinan University, China. Well-ripened dry date palm fruits (cultivar: Rubai) were washed and surface dried with a paper towel. The date palm fruit had a high-sugar content of 81% with 3.2% fiber and 0.3% pectin. The pits in the fruits were removed, and the fruits were cut into small pieces. About 100 g of cut pieces were mixed with 300-ml distilled water and boiled for 30 min. The mixture was then cooled to room temperature and homogenized. The homogenate was depectinized under predetermined optimized conditions: pectinase, 48 U/g; incubation temperature, 40°C for 38 min. After depectinization, the mixture was boiled again for 10 min to inactivate the pectinase, cooled to room temperature on ice, and then mixed with 0.036% NaHSO_3_ and ACTIFLORE F33 yeast (0.03%, Laffort, France) for fermentation, which proceeded anaerobically at 28°C for 8 days. The sugar content was determined and recorded every day, using a refractometer (Mettler Toledo, Leicester, USA). The initial sugar content before fermentation was 27 °Brix, the final sugar content of wine was 7.3°Brix, and the alcohol content was ~15%. The total folate and distribution of folate vitamers in the wine were determined daily. Subsequently, the wine was packed in separate tubes and stored at −80°C until further folate determination.

### Folate Analysis in Fermentation Reagents

To take into consideration folate brought by the fermentation reagents, the endogenous folate content in the fermentation reagents was determined as suggested in early reports (10, 18). Specifically, 5-g samples of fermentation reagents (pectinase and ACTIFLORE F33 yeast) were suspended in 20 ml of an extraction buffer (100-mM ammonium acetate; 1% ascorbic acid; 0.2% 2-mercaptoethanol; pH, 7.89) and homogenized for 2 min using a homogenizer (IKA T18 Basic; IKA, Staufen, Germany) at 4°C. The homogenates were immediately boiled for 15 min, cooled on ice to room temperature, and reconstituted to 25 ml. After this, the extraction was carried out as detailed earlier, and further hydrolysis was carried out as follows.

### Folate Deconjugation With Recombinant Human GGH, SPE Cleanup, and UHPLC-MS/MS Determination of Folate Vitamers and Total Folate

For the past two decades, protease and amylase have been applied during folate extraction to improve folate release from the food matrix into the extraction buffer and to determine the concentration of folate vitamers and total folates in a high-protein or starchy food matrix (19). For yeast and pectinase extract, 1.5 ml of pronase (10 mg/ml) was added to 25 ml of the extraction, followed by incubation at 37°C for 3 h. After treatment with pronase, 1.5 ml of α-amylase was added and incubated again at 37°C for 2 h. The pronase- and amylase-treated yeast extract and the date palm fruit wine were subjected to the following deconjugation. As pointed out earlier, intracellular folates can be found in the form of polyglutamates, and folate conjugase can be used to hydrolyze them into monoglutamyl forms. To determine individual folate vitamers in different samples, 5-ml aliquots of different extracts of date palm fruit extract or fermentation reagents (pectinase and ACTIFLORE F33 yeast) or 20 ml of fermented wine were treated with recombinant GGH (5 or 1 μg/ml) at 37°C for 30 min to deconjugate polyglutamyl folates to its monoglutamyl forms. After incubation, all samples were boiled for 5 min and centrifuged at 20,000 rpm for 10 min. The supernatant was recovered and subjected to solid-phase extraction (SPE). The completeness of the deconjugation reaction was determined by monitoring the profile of polyglutamyl folate and the total folate contents (6).

Solid-phase extraction was carried out before UHPLC-MS/MS analysis to remove the interferences and decrease the matrix effect of water-soluble components, such as sugar. Oasis HLB6 cc Vac cartridges (Waters, Milford, MA) were first activated and conditioned successively with 5 ml of methanol and 5 ml of water. Before application to the SPE cartridge, deconjugated extracts were adjusted to pH 2 with 6 mol/L HCl. After the sample was applied, the cartridge was washed with 5 ml of 5% methanol in water and evaporated to dryness in a vacuum. Folate was eluted with 5 ml of 95% acetonitrile in 5% methanol. Finally, the eluate was evaporated to dryness in a stream of nitrogen, reconstituted with 1 ml of the extraction buffer, and filtered through a 0.22-μm nylon filter before injection into the UHPLC-MS/MS system.

Quantitation of folate vitamers was carried out, following the methods described in Zou et al. (6). UHPLC separation was performed with an Agilent 1290 Infinity UHPLC equipped with a binary pump, an autosampler, a column oven, and degasser. The column was a HILIC column (4.5 × 100 mm, 2.6 μm, Kinetex, Phenomenex), and its temperature was set at 40°C. The mobile phase was 0.1% formic acid in water (A) and 0.1% formic acid in acetonitrile (B). Its flow rate was 0.8 ml/min. The gradient was as follows: 0–4 min, 0–20% B; 4–5 min, 20–95% B; 5–6.5 min, 95% B; 6.5–9 min, 0% B. The injection volume was 2 μl, and the autosampler was kept at 25°C. The UHPLC eluate was introduced into an Agilent 6460 triple quadrupole mass spectrometer. The MS/MS instrument was operated in the positive-ion electrospray mode at a capillary voltage of +3.5 kV, and a charging voltage of +1 kV. Nitrogen was used as the nebulizing gas at 0.31 MPa along with a carrier gas flowing at 10 L/min at 300°C and a sheath gas flowing at 11 L/min at 350°C. An Agilent Mass Hunter workstation was used to control the equipment for data acquisition and analysis. Acquisitions were performed by selected reaction monitoring.

### Statistical Analysis

Microsoft Excel was used to compute the mean ± standard deviation (SD) concentrations of each folate form. Significant differences were determined by *ANOVA*, using Tukey's *post-hoc* test and a paired *t*-test using Minitab 19.0 (State College, PA). A significant difference between the means was defined as *p* < 0.05.

## Results and Discussion

### Impact of Individual Factors on Pectinase-Aided Folate Extraction Efficiency

**Figure 3:**
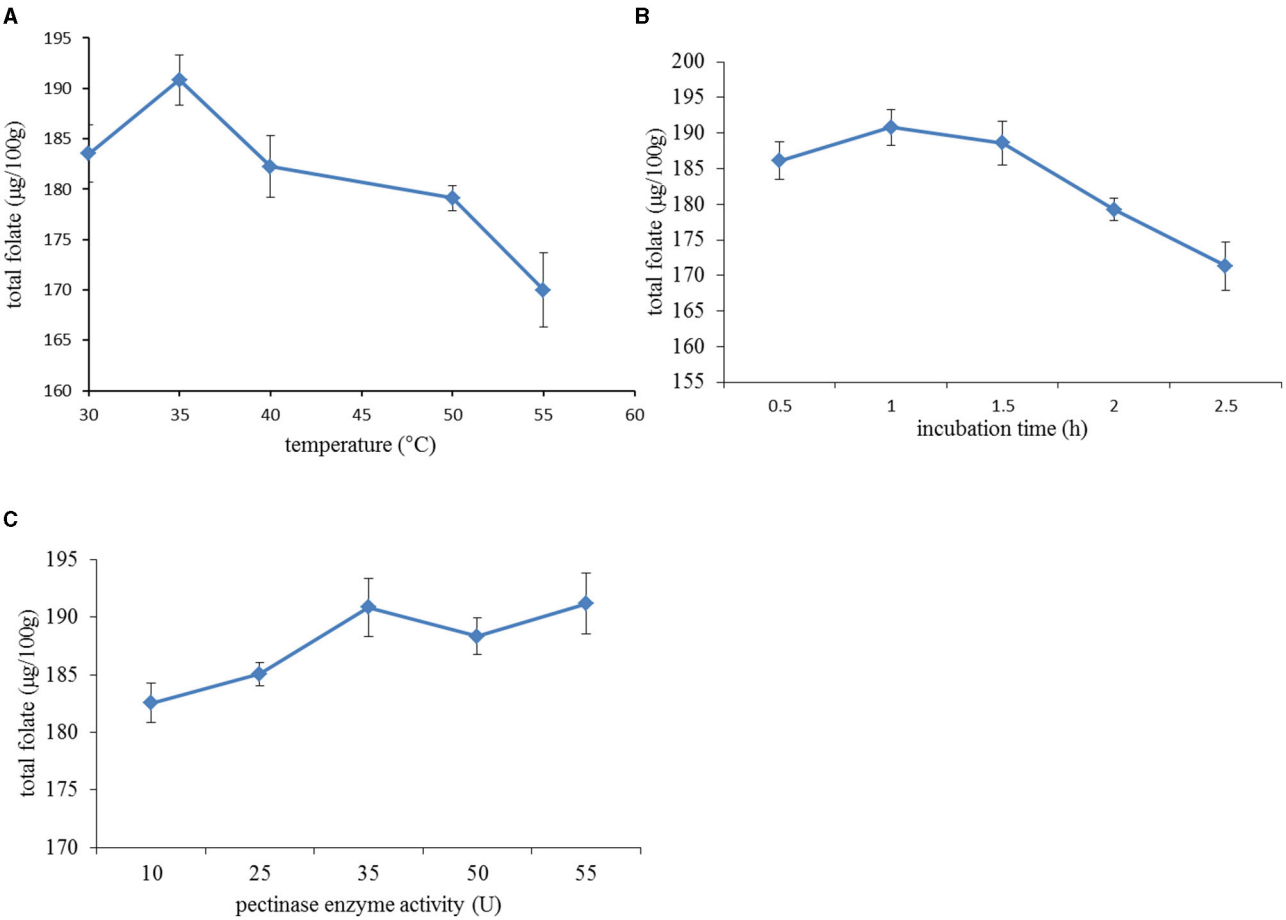
Effect of different extraction variables on total folate content: **(A)** extraction temperature: the incubation time and pectinase concentration were fixed at 1 h and 35 U/g; **(B)** incubation time: incubation temperature and pectinase concentration were set at 35°C and 35 U/g; **(C)** pectinase enzyme activity: the incubation temperature and time were maintained at 35°C and 1 h.

Three factors, namely, pectinase concentration, incubation time, and incubation temperature were tested as independent factors in this study to identify their importance in the extraction of folate from date palm fruit extract. The effect of incubation temperature (first variable tried for optimization) on the folate extraction yield is shown in Figure 3A with the yield positively correlating with increasing incubation temperature from 30 to 35°C; however, it then decreased when the temperature was above 35°C. Although the supporting documents from the manufacturer suggested that pectinase activity was optimum at 45°C, it was observed that total folate actually decreased when temperature was above 35°C. This phenomenon could be attributed to the folate degradation induced by higher incubation temperatures beyond 40°C (20, 21). According to an early report, folates are very sensitive molecules and can degrade ~10–20% after incubation at 37°C for 2 h, which leads to the cleavage of folate into a pteridine and *p*-aminobenzoic acid linked to a polyglutamate tail (22). Neither pteridine nor *p*-aminobenzoic acid has biological functions. This optimum level of 35°C was used when testing the other two factors.

The extraction yields at different incubation times (second variable) are shown in Figure 3B, which increased with incubation time between 0.5 and 1.5 h and decreased at longer treatment times. The initial increase in folate markedly increased with the extension of incubation time, which was attributed to the gradual decrease in resistance to intracellular folate diffusion during pectin hydrolysis. Still, longer incubation time caused the degradation of folate due to its thermal susceptibility. Also, there was no significant difference when the extract was incubated with enzyme for 1 and 1.5 h. Therefore, the incubation time was set at 1 h to test the effect of pectinase concentration on the extraction yield.

The influence of pectinase concentration was finally tested on the extraction yield and is detailed in Figure 3C, which, like before, showed that the total folate yield increased rapidly with increasing concentration of pectinase up to a middle level of 35 U/g; after which, the further increases were not significant. Hence, a 35 U/g enzyme concentration was considered appropriate. This effect could be explained by the fact that a high pectinase concentration promoted the decomposition of pectin in the cell walls of date palm fruits, which contributed to enhance the permeability of cell membranes and the solubility of folate. It is well-known that pectinase helps to increase the juice yield by degrading cell wall polysaccharides and improving the compressibility of pulp for extraction (23).

### RSM Optimization of Pectinase-Assisted Extraction of Folate

In the previous section, three test factors in folate extraction were evaluated over a broader span but without considering their interactions. In the past, that was one of the optimization pathways, achieving the goal in a stepwise fashion. Such an approach is simple and good for identifying suitable conditions where the factors could influence the independent output. However, to be meaningful, the interactive effects of the different factors need to be considered because they can have synergistic (positive) or antagonistic (negative) effects. An RSM approach is generally used for this purpose. A three-factor, three-level RSM experimental design was used with the central level at the near-optimum conditions as determined from the previous experiments. The levels of the three factors used [pectinase concentration (X_1_), incubation temperature (X_2_), and incubation time (X_3_)] are detailed in Table 3.

The effects and interactions for each variable were optimized by BBD. A second-degree polynomial equation was developed as indicated by the following equation as the relationship between the total folate content and coded factors (Table 4):

(2) $$\begin{array}{r} \text{Total folate content }\left(\text{μg}/100\text{g}\right)=-11.4+1.165{\text{X}}_{1}+6.41{\text{X}}_{2}\\ \;+147.80{\text{X}}_{3}-0.02464{\text{X}}_{1}^{2}\\ \;-0.0905{\text{X}}_{2}^{2}-29.05{\text{X}}_{3}^{2}\\ \;+0.04191{\text{X}}_{1}{\text{X}}_{2}-0.7874{\text{X}}_{1}{\text{X}}_{3}\\ \;-1.842{\text{X}}_{2}{\text{X}}_{3} \end{array}$$

The analysis of variance (ANOVA) results applied to validate the regression equation and confirm the influence of each factor on total folate content. The results are shown in Table 4. The *P* and *F*-values are used to evaluate the importance of each parameter. Low *P* and high *F-*values indicated that the experimental factors were highly significant (24). The relationship between the above regression equation and the response surface was highly significant (*P* = 0.000). Additionally, all of the factors were extremely significant variables (*p* ≤ 0.01) (Table 4) except X_1_. This was probably because its individual effects were confounded by the interactions with the other two factors, and they were highly significant; therefore, the X_1_ factor is still a significant player in the overall model. In addition, three factors had extremely significant quadratic effects on the extraction of folate (*p* < 0.01). The *F*-value of the model was 115.27, indicating that model was statistically significant. The relativity between lack of fit and pure error is significant because the “lack-of-fit *F*-value” was not significant. The high *R*^2^-value (*R*^2^ = 0.93) indicated that the predicted values compared well with the experimental values. *R*^2^ will be slightly increased if all variables and their interaction terms were included in the model regardless whether it was statistically significant or not. An adjusted *R*^2^ ($R_{\text{Adj}}^{2}$) was used instead of *R*^2^ because $R_{\text{Adj}}^{2}$ would not change a given variable when the interaction term was added or deleted. In this model, an $R_{\text{Adj}}^{2}$ of 0.99 was obtained, revealing that 99% of the total variations were explained by the model. Consequently, the regression equation could well-reflect the real relationship between experimental factors and the total folate and could be employed to obtain the optimal extraction parameters for the extraction of folate from date palm fruits.

**Table 4:** Results of *ANOVA* of the regression equation.

**Source**	**DF**	**Adj SS**	**Adj MS**	***F*** **-value**	***P*** **-value**
Model	9	496.829	55.203	115.27	0.000
X_1_	1	0.013	0.013	0.03	0.877
X_2_	1	7.876	7.876	16.45	0.010
X_3_	1	36.748	36.748	76.74	0.000
${\text{X}}_{1}^{2}$	1	54.727	54.727	114.28	0.000
${\text{X}}_{2}^{2}$	1	18.898	18.898	39.46	0.002
${\text{X}}_{3}^{2}$	1	194.786	194.786	406.75	0.000
X_1_ X_2_	1	27.448	27.448	57.32	0.001
X_1_ X_3_	1	96.886	96.886	202.32	0.000
X_2_ X_3_	1	84.788	84.788	177.05	0.000
Error	5	2.394	0.479		
Lack of fit	3	2.193	0.731	7.25	0.124
Pure error	2	0.202	0.101		
Total	14	499.223			

**Figure 4:**
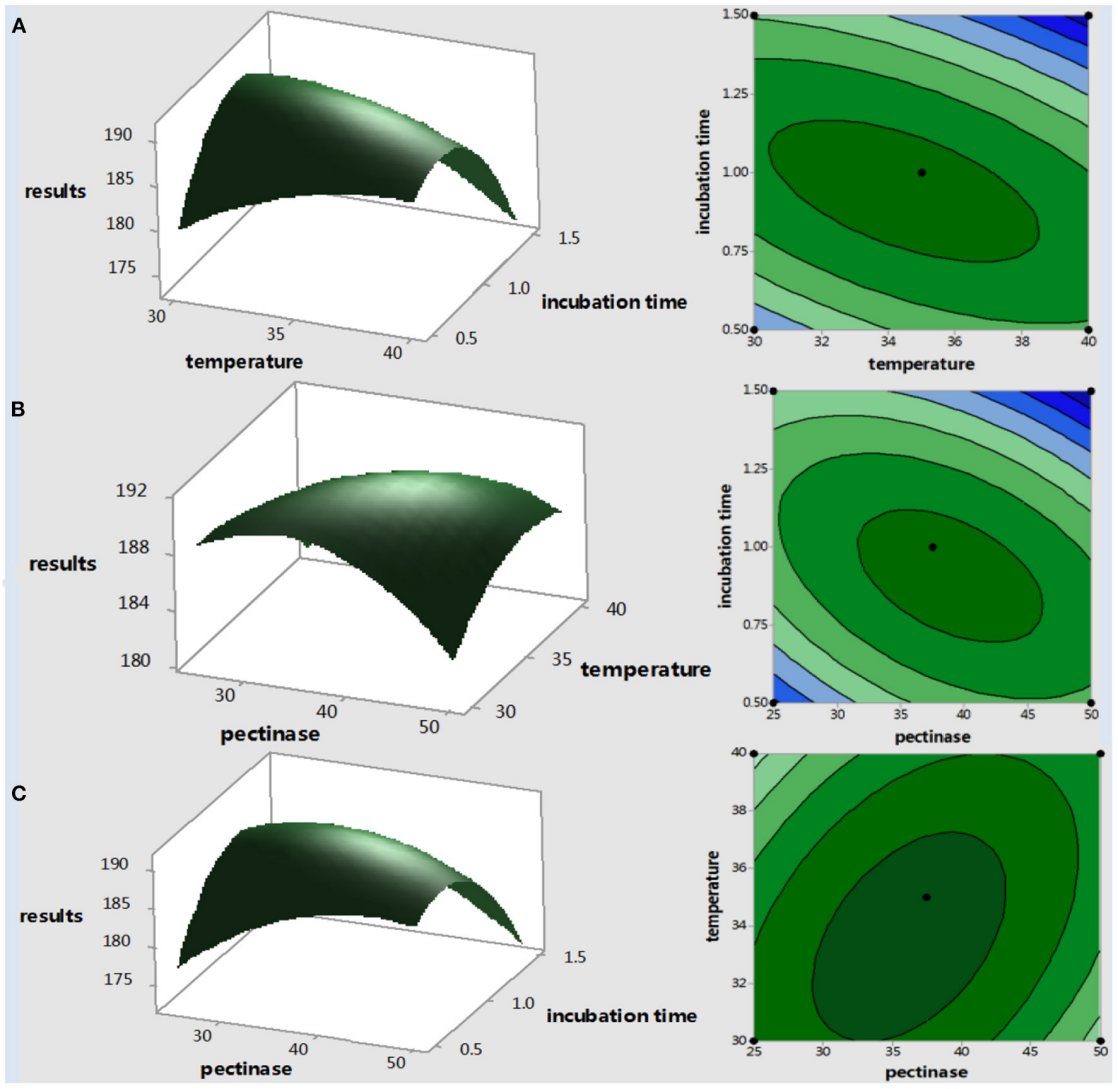
Response surfaces and contour plots for the effect of **(A)** incubation temperature (X_2_) and incubation time (X_3_), **(B)** pectinase enzyme activity (X_1_), and extraction temperature (X_2_) **(C)** pectinase enzyme activity (X_1_) and incubation time (X_3_).

Three-dimensional surface plots and two-dimensional contour plots are presented to visualize general variation trends between operational parameters, and the shapes of the response surface curves indicated significant interactions between the extraction variables and the yield parameters (Figure 4), as done in other studies (25). In these 3-D plots, the influence of two variables is shown by keeping the third variable at its central level (because it is not possible to show the fourth dimension). 3D response surfaces (Figure 4) show a convex shape, which means that the ranges used for extraction variables are appropriate, and an optimal shape can be obtained within the range.

An early study found that steep 3D response surface plots show that the experimental factors have a great influence on the extraction of total folate, and the interaction of the two experimental factors is highly related (23). Additionally, early studies have implicated that, in the 2D contour, the oval and circular contours reveal the interaction between the two factors is significant and not significant, respectively (23). Here, in this study, the 2D contour plots were all oval, especially the interactions between X_2_ and X_3_, X_1_ and X_2_ were more significant than X_1_ and X_3_, which is consistent with the *ANOVA* results in Table 4.

Through the above data analysis, the optimized conditions were obtained as follows: pectinase activity: 47.5 U, incubation temperature of 40°C, and incubation time of 38 min. Experimental rechecking was performed to compare the predicted total folate with the experimental value evaluated under the optimal processing conditions. For this experiment, the best extraction processing conditions were rounded as follows: pectinase activity: 48 U, incubation temperature of 40°C, and incubation time of 38 min. The results showed that, under these conditions, total folate reached 193 ± 3 μg/100 g, which is close to the predicted total folate of 191 μg/100 g (error within 1%).

Response surface methodology has also been used in other studies involving folate extraction. For example, the trienzyme extraction of folate from different starchy or high-protein foods was optimized by RSM, demonstrating a shorter extraction time and minimization of folate loss (16, 17). However, this is the first study to optimize RSM for folate extraction from fruit with a high-pectin content by the application of pectinase, while some previous studies have hinted that pectin and oligosaccharides or polysaccharides could entrap and slow the release of folate (8, 9). Furthermore, the optimized method could possibly be applied for folate determination in some high-pectin vegetables and fruits, such as carrots, strawberries, gourds, and many others.

### Folate Vitamer Distribution and Total Folate in Different Date Palm Cultivars by Optimized RSM Extraction

**Figure 5:**
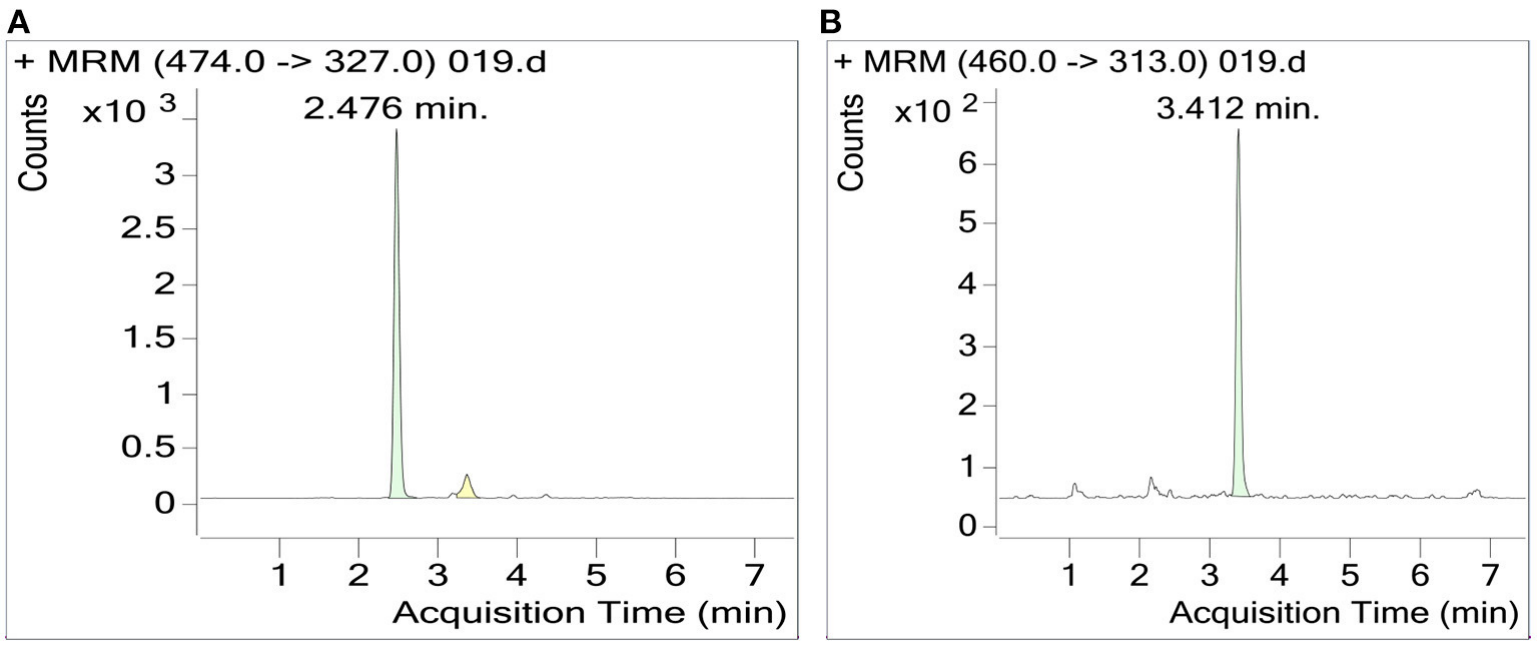
UHPLC-MS/MS chromatogram of 5-CHO-THF **(A)** and 5-CH_3_-THF **(B)**. The MS/MS transition of 5-CHO-THF and 5-CH_3_-THF: 474 > 327; 460 > 313, respectively.

The RSM-optimized method was applied for folate extraction from three cultivars of date palm fruit cultivars: Zahdi, Rubai, and Muzafti. In all of them, 5-CHO-THF was the predominant form of folate vitamer, accounting for 91–97%, and 5-CH_3_-THF was a minor vitamer, accounting for 3–9% of the total folates. The UHPLC-MS/MS chromatogram is shown in Figure 5. The total folate contents were found to be 191 (±2.52), 229 (±3.80), and 301 (±2.29) μg/100 g for Zahdi, Rubai, and Muzafti, respectively. According to Institute of Medicine, the recommended dietary allowance (RDA) of folate is 400 μg per day (26). Thus, serving 100 g of date fruits (without seeds) would provide 48, 57, and 75% of the total folate, respectively, with the cultivar Muszafti contributing the most. In general, all date palm fruits investigated have high-folate content and can use as supplements for folate nutrition. Different folate vitamers and total folate contents in date palm fruits have never been characterized before; therefore, this is valuable information (27).

### Total Folate Tracking During Date Palm Fruit Fermentation

**Figure 6:**
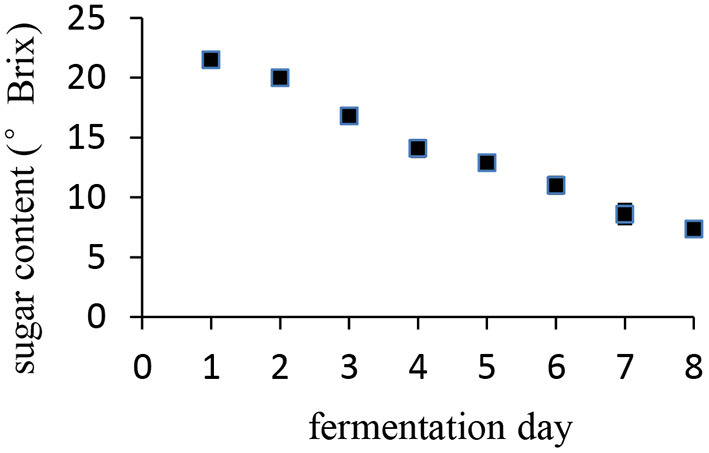
Change of sugar content during date palm wine making.

Date palm fruits are a staple food, and they are not only eaten raw but are also fermented into wine. The course of the date palm fruit fermentation process is shown in Figure 6. The sugar content of the syrup decreased during fermentation until from 27°Brix to 7.3°Brix in 8 days, resulting in ~15% alcohol. The relationship between sugar content and the fermentation day was almost linear.

**Figure 7:**
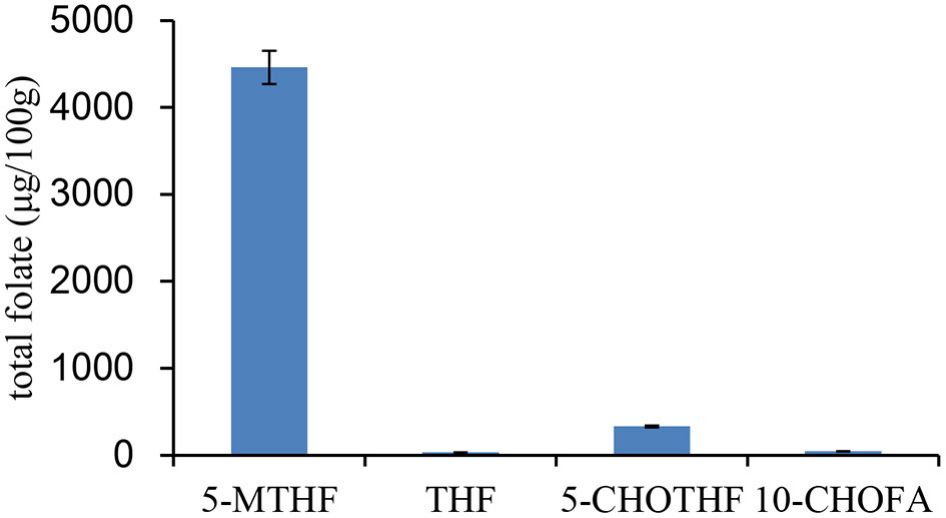
The distribution of folate vitamer and total folate content in brewer's yeast.

The folate vitamer and total folate in pectinase and brewer's yeast were first evaluated (Figure 7). The results showed that there were four types of folate vitamers in brewer's yeast: 5-CH_3_-THF (4,460 ± 191 μg/100 g), 5-CHO-THF (330 ± 12 μg/100 g), 10-CHOFA (45 ± 2 μg/100 g), and THF (31 ± 1 μg/100 g). The total folate content in the yeast was 4,870 μg/100 g. The yeast used in the study is the common strain used in grape wine fermentation. This is the first report on folate vitamers and total folate in brewer's yeast; most other studies reported it on bakers' yeasts. Brewer's yeast contains higher total folate than the baker's yeast, which is usually reported to be ~4,000 μg/100 g (18, 28). However, the distribution of folate vitamers in brewer's yeast was similar to that in baker's yeast, which also had 5-CH_3_-THF as the predominant form (92%), followed by 5-CHO-THF (6.8%) and minor constituents of THF and 10-CHOFA. Therefore, in general, yeast is a good source of folate. Additionally, endogenous folate in pectinase was too low to be detected.

**Figure 8:**
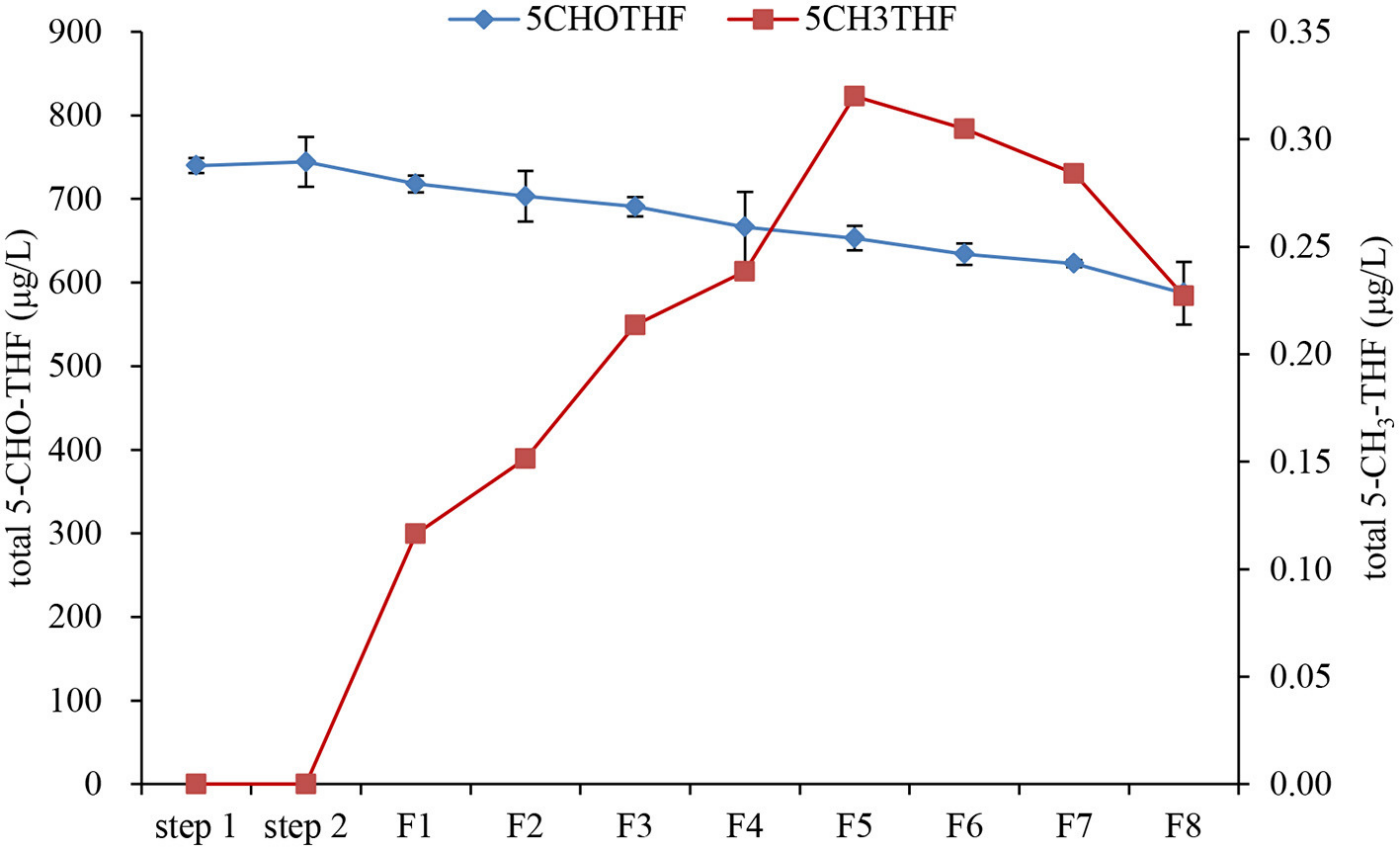
Further changes of folate vitamers and total folate during fermentation step 1: pectinase hydrolysis; step 2: inactivation of pectinase after hydrolysis; F1 to F8: fermentation day 1 to day 8.

Further changes in folate vitamers and total folate during fermentation were also evaluated daily (Figure 8). The results clearly showed that there were two types of folate vitamers in date palm wine: 5-CHO-THF and 5-CH_3_-THF. During fermentation, the content of 5-CHO-THF gradually decreased from day one, resulting in a total decrease of ~20% after fermentation for 8 days, although 5-CHO-THF is the most stable form of natural folate. The significant loss of 5-CHO-THF during wine making could be because, during yeast proliferation, some enzymes or compounds might be produced that could lead to 5-CHO-THF degradation. However, this has never been reported earlier. In future studies, the mechanism of 5-CHO-THF degradation could be explored. Additionally, there was a significant increase in 5-CH_3_-THF during fermentation from day 1 to day 5. This increase followed linear rate kinetics (y = 0.058x + 0.0284, *R*^2^ = 0.9614). The increased 5-CH_3_-THF supposedly came from the growth of yeast. 5-CH_3−_THF is an important folate derivative that plays a key role as a methyl donor in the regeneration of methionine from homocysteine, produced in the methylation cycle of one carbon (7). Several studies have reported the biofortification of folate in different food systems with baker's yeast (29). Hjortmo et al. (30) detected 3–5-fold higher total folate in white bread leavened with *Saccharomyces cerevisiae* strain CBS7764 compared to commercial yeast. Walkey et al. (31) reported the enhancement of folate levels in wine by bioengineering the yeast and found that the folate level in wine was mainly contributed by the yeast used, with strain dependence (31). *Saccharomyces cerevisiae* has the ability to synthesize folates because of its genetic apparatus (32). During folate biosynthesis by yeast, three major genes FOL2, FOL3, and DFR1, which encode three important enzymes, were identified (32). GTP-cyclohydrolase I, encoded by FOL2, is the first enzyme in the folate biosynthesis pathway. Dihydrofolate synthase, encoded by FOL3, produces dihydrofolate, while dihydrofolate reductase, encoded by DFR1, converts dihydrofolate into tetrahydrofolate. Tetrahydrofolate will then interconvert to 5-CH_3_-THF in one-carbon transfer reactions (32). In addition to yeast, other fermented foods can also biosynthesize folate. D'Aimmo et al. (33) screened 19 strains of bifidobacteria for different folate forms and total folate and found that most strains had a total folate content above 4,000 μg/100 g dry matter, demonstrating that bifidobacteria may contribute to folate intake by applying suitable growth conditions (33). Additionally, some lactic acid bacteria (LAB) species are able to produce folates in fermented milk (34). Moreover, cocultures of different LAB species have been used to increase the content of folates by ~30% compared to single cultures (35). All of these studies have proved that food fermentation could be a useful tool to increase total folate intake. Furthermore, the stability of folate during date palm fruit wine fermentation must also be considered to properly evaluate the folate content since the degradation and interconversion of folate vitamers can have a deep impact on the final concentration of folates (35). After fermentation, the total folate in the wine reached a maximum level of 700 μg/L. Thus, it was demonstrated that when date palm fruit was processed into wine, total folate was conserved and the sugar content and calories drastically decreased.

## Conclusions

The pectinase-mediated facilitation of the extraction of folate from date palm fruits was characterized using UHPLC-MS/MS analysis. This is the first such study on folate extraction from date palm fruits, demonstrating the influence of the principal variables, pectinase activity, incubation temperature, and incubation time for efficient release of folate from the pectin matrix. It is suggested that, for other fruits and vegetables containing high levels of pectin, pectinase should be considered for improving the extraction efficiency of folate as a cost-effective means of folate extraction.

By applying an optimized RSM methodology, folate vitamer distribution, and total folate content in three cultivars of date palm fruits (Muzafti, Zahdi, and Rubai) were determined. This is the first study to demonstrate that date palm fruits are good sources of dietary folate due to their high-folate content (191–301 μg/100 g) and will add a new ingredient to the USDA Nutrient Database.

Changes in folate vitamers and total folate during date palm fruit wine fermentation were tracked. Two types of folate vitamers were identified as predominant 5-CHO-THF and minor 5-CH_3_-THF. Interestingly, during fermentation, 5-CHO-THF decreased by ~20%, while 5-CH_3_-THF first increased from 0 to 0.32 μg/L and then significantly decreased. Further studies are needed to understand the mechanism of the loss of 5-CHO-THF and the generation of 5-CH_3_-THF. Additionally, a new strategy should be designed to improve folate stability during fermentation and storage.

## Data Availability Statement

The original contributions presented in the study are included in the article/supplementary material, further inquiries can be directed to the corresponding author/s.

## Author Contributions

ZM and LY: methodology, formal analysis, investigation, and writing–original draft preparation. QH and ZL: validation. HR: conceptualization, writing—review, editing, and supervision. CW: conceptualization, writing—review and editing, supervision, and funding acquisition. All authors contributed to the article and approved the submitted version.

## Funding

This research was supported by Project of Science and Technology of Guangzhou, (Grant No. 201804010367) and Key-Area Research and Development Program of Guangdong Province (No. 2020B020225003).

## Conflict of Interest

The authors declare that the research was conducted in the absence of any commercial or financial relationships that could be construed as a potential conflict of interest.

## Publisher's Note

All claims expressed in this article are solely those of the authors and do not necessarily represent those of their affiliated organizations, or those of the publisher, the editors and the reviewers. Any product that may be evaluated in this article, or claim that may be made by its manufacturer, is not guaranteed or endorsed by the publisher.

## References

[1] Stover PJ. Polymorphisms in 1-carbon metabolism, epigenetics and folate-related pathologies. J Nutrigenet Nutrige. (2011) 4:293–305. 10.1159/000334586 22353665 PMC3696357

[2] Busso D Echeverría G Passi-Solar A Morales F Farías M Margozzini P. Folate status in women of childbearing age in the Urban Metropolitan Region of Chile: results from the National Health Survey 2016-2017. Public Health Nutr. (2021) 24:385–92. 10.1017/S1368980020002608 32907649 PMC7844604

[3] Strobbe S Van Der Straeten D. Folate biofortification in food crops. Curr Opin Biotechnol. (2017) 44:202–11. 10.1016/j.copbio.2016.12.003 28329726

[4] Scott J Rébeillé F Fletcher J. Folic acid and folates: the feasibility for nutritional enhancement in plant foods. J Sci Food Agric. (2000) 80:795–824. 10.1002/(SICI)1097-0010(20000515)80:7<795::AID-JSFA599>3.0.CO;2-K

[5] Hou SY Man XX Lian BY Ma GF Sun ZX Han LD. Folate metabolic profiling and expression of folate metabolism-related genes during panicle development in foxtail millet (Setaria italica (L.) P. *Beauv*). J Sci Food Agric. (2021). [Epub ahead of print]. 10.1002/jsfa.11355 34109642

[6] Zou YC Duan HY Li L Chen XJ Wang C. Quantification of polyglutamyl 5-methyltetrahydrofolate, monoglutamyl folate vitamers, and total folates in different berries and berry juice by UHPLC-MS/MS. Food Chem. (2019) 276:1–8. 10.1016/j.foodchem.2018.09.151 30409571

[7] Saini RK Nile SH Keum Y-S. Folates: chemistry, analysis, occurrence, biofortification and bioavailability. Food Res Int. (2016) 89:1–13. 10.1016/j.foodres.2016.07.013 28460896

[8] Cavalcanti Albuquerque MA Yamacita DS Bedani R LeBlanc JG Saad SMI. Influence of passion fruit by-product and fructooligosaccharides on the viability of streptococcus thermophilus TH-4 and lactobacillus rhamnosus LGG in folate bio-enriched fermented soy products and their effect on probiotic survival and folate bio-accessibility under *in vitro* simulated gastrointestinal conditions. Int J Food Microbiol. (2019) 292:126–36. 10.1016/j.ijfoodmicro.2018.12.012 30597427

[9] Estevinho BN Lazar R Blaga A Rocha F. Preliminary evaluation and studies on the preparation, characterization and *in vitro* release studies of different biopolymer microparticles for controlled release of folic acid. Powder Technol. (2020) 369:279–88. 10.1016/j.powtec.2020.05.048

[10] Luo SY Duan HY Zu YC Qiu RX Wang C. Quantification of total folate, folate species and polyglutamyl folate distribution in winged beans (Psophocarus tetragonolobus (L) DC) from different cultivars and growth stages by ultra-high performance liquid chromatography tandem mass spectrometry. J Nutr Sci Vitaminol. (2017) 63:69–80. 10.3177/jnsv.63.69 28367928

[11] Aslam J Khan SH Khan SA. Quantification of water soluble vitamins in six date palm (Phoenix dactylifera L.) cultivar's fruits growing in Dubai, United Arab Emirates, through high performance liquid chromatography. J Saudi Chem Soc. (2013) 17:9–16. 10.1016/j.jscs.2011.02.015

[12] Al-Harrasi A Rehman NU Hussain J Khan AL Al-Rawahi A Gilani SA. Nutritional assessment and antioxidant analysis of 22 date palm (Phoenix dactylifera) varieties growing in Sultanate of Oman. Asian Pac J Trop Med. (2014) 7:S591–8. 10.1016/S1995-7645(14)60294-7 25312188

[13] Parvin S Easmin D Sheikh A Biswas M Sharma SCD Jahan MGS. Nutritional analysis of date fruits (Phoenix dactylifera L.) in perspective of Bangladesh. Am J Life Sci. (2015) 3:274–8. 10.11648/j.ajls.20150304.14

[14] Al-Alawi RA Al-Mashiqri JH Al-Nadabi JSM Al-Shihi BI Baqi Y. Date palm tree (Phoenix dactylifera L.): natural products and therapeutic options. Front Plant Sci. (2017) 8:845. 10.3389/fpls.2017.00845 28588600 PMC5440559

[15] Matloob MH. Zahdi date vinegar: production and characterization. Am J Food Tech. (2014) 9:231–45. 10.3923/ajft.2014.231.245

[16] Cho S Choi Y Lee J Eitenmiller RR. Optimization of enzyme extractions for total folate in cereals using response surface methodology. J Agric Food Chem. (2010) 58:10781–6. 10.1021/jf102751w 20843040

[17] Choi Y-M Eitenmiller RR Kim S-H Lee J-S. Optimization of tri-enzyme extraction procedures for the microbiological assay of folate in red kidney bean and roasted peanut using response surface methodology. Food Sci Biotechnol. (2009) 18:31–5. 10.1016/j.foodpol.2008.07.001

[18] Gmelch L Wirtz D Witting M Weber N Striegel L Schmitt-Kopplin P. Comprehensive vitamer profiling of folate mono- and polyglutamates in baker's yeast (Saccharomyces cerevisiae) as a function of different sample preparation procedures. Metabolites. (2020) 10:301. 10.3390/metabo10080301 32717862 PMC7464241

[19] Wang C Riedl KM Schwartz SJ. A liquid chromatography-tandem mass spectrometric method for quantitative determination of native 5-methyltetrahydrofolate and its polyglutamyl derivatives in raw vegetables. J Chromatogr B. (2010) 878:2949–58. 10.1016/j.jchromb.2010.08.043 20888309 PMC3850028

[20] Wang C Riedl KM Schwartz SJ. Fate of folates during vegetable juice processing - deglutamylation and interconversion. Food Res Int. (2013) 53:440–8. 10.1016/j.foodres.2013.05.011

[21] Aprodu I Dumitraşcu L Râpeanu G Bahrim GE Stănciuc N. Spectroscopic and molecular modeling investigation on the interaction between folic acid and bovine lactoferrin from encapsulation perspectives. Foods. (2020) 9:744. 10.3390/foods9060744 32512783 PMC7353600

[22] Brouwer VD Zhang G-F Storozhenko S Van Der Straeten D Lambert WE. pH stability of individual folates during critical sample preparation steps in prevision of the analysis of plant folates. Phytochem Anal. (2007) 18:496–508. 10.1002/pca.1006 17624887

[23] Xue HK Tan JQ Li Q Tang JT Cai X. Ultrasound-assisted enzymatic extraction of anthocyanins from raspberry wine residues: process optimization, isolation, purification, and bioactivity determination. Food Anal Method. (2021) 14:1369–86. 10.1007/s12161-021-01976-8

[24] Liu Y Wei SL Liao MC. Optimization of ultrasonic extraction of phenolic compounds from Euryale ferox seed shells using response surface methodology. IND Crop Prod. (2013) 49:837–43. 10.1016/j.indcrop.2013.07.023

[25] Vajic U-J Grujic J Živković J Savikin K Godevac D Miloradović Z Bugarski B. Optimization of extraction of stinging nettle leaf phenolic compounds using response surface methodology. Ind Crop Prod. (2015) 74:912–7. 10.1016/j.indcrop.2015.06.032

[26] Finglas P. Dietary reference intakes for thiamin, riboflavin, niacin, vitamin B6, folate, vitamin B12, pantothenic acid, biotin, and choline. Trends Food Sci Tech. (2000) 11:296–7. 10.1016/S0924-2244(01)00010-3 23193625

[27] Al-Orf SM Ahmed MHM Al-Atwai N Al-Zaidi H Dehwah A Dehwah S. Review: nutritional properties and benefits of the date fruits (Phoenix dactylifera L.). Bull Natl Nutr Instit Arab Republic Egypt. (2012) 39:98–129.

[28] Patring JDM Jastrebova JA Hjortmo SB Andlid TA Jägerstad IM. Development of a simplified method for the determination of folates in baker's yeast by HPLC with ultraviolet and fluorescence detection. J Agric Food Chem. (2005) 53:2406–11. 10.1021/jf048083g 15796570

[29] Rai AK Pandey A Sahoo D. Biotechnological potential of yeasts in functional food industry. Trends Food Sci Technol. (2019) 83:129–37. 10.1016/j.tifs.2018.11.016

[30] Hjortmo S Patring J Jastrebova J Andlid TA. Biofortification of folates in white wheat bread by selection of yeast strain and process. Int J Food Microbiol. (2008) 127:32–6. 10.1016/j.ijfoodmicro.2008.06.001 18599142

[31] Walkey CJ Kitts DD Liu YZ van Vuuren HJJ. Bioengineering yeast to enhance folate levels in wine. Process Biochem. (2015) 50:205–10. 10.1016/j.procbio.2014.12.017

[32] Goncerzewicz A Misiewicz A. The sequence diversity and expression among genes of the folic acid biosynthesis pathway in industrial Saccharomyces strains. Acta Biochim Pol. (2015) 62:841–50. 10.18388/abp.2015_1144 26610311

[33] D'Aimmo MR Modesto M Mattarelli P Biavati B Andlid T. Biosynthesis and cellular content of folate in bifidobacteria across host species with different diets. Anaerobe. (2014) 30:169–77. 10.1016/j.anaerobe.2014.09.018 25312826

[34] Revuelta JL Serrano-Amatriain C Ledesma-Amaro R Jiménez A. Formation of folates by microorganisms: towards the biotechnological production of this vitamin. Appl Microbiol Biotechnol. (2018) 102:8613–20. 10.1007/s00253-018-9266-0 30073396 PMC6153639

[35] Saubade F Hemery YM Guyot J-P Humblot C. Lactic acid fermentation as a tool for increasing the folate content of foods. Crit Rev Food Sci Nutr. (2017) 57:3894–910. 10.1080/10408398.2016.1192986 27351520

